# Evolutionary bet-hedging in structured populations

**DOI:** 10.1007/s00285-021-01597-z

**Published:** 2021-04-01

**Authors:** Christopher E. Overton, Kieran J. Sharkey

**Affiliations:** 1 University of Liverpool, Liverpool, UK; 2 University of Manchester, Manchester, UK

**Keywords:** Evolution, Fitness variance, Networks, Evolutionary graph theory, Stochastic process, 92-10, 92D15, 92D40

## Abstract

As ecosystems evolve, species can become extinct due to fluctuations in the environment. This leads to the evolutionary adaption known as bet-hedging, where species hedge against these fluctuations to reduce their likelihood of extinction. Environmental variation can be either within or between generations. Previous work has shown that selection for bet-hedging against within-generational variation should not occur in large populations. However, this work has been limited by assumptions of well-mixed populations, whereas real populations usually have some degree of structure. Using the framework of evolutionary graph theory, we show that through adding competition structure to the population, within-generational variation can have a significant impact on the evolutionary process for any population size. This complements research using subdivided populations, which suggests that within-generational variation is important when local population sizes are small. Together, these conclusions provide evidence to support observations by some ecologists that are contrary to the widely held view that only between-generational environmental variation has an impact on natural selection. This provides theoretical justification for further empirical study into this largely unexplored area.

**Supplementary Information:**

The online version supplementary material available at 10.1007/s00285-021-01597-z.

## Introduction

Many traits in biological populations have been explained by selection for risk-spreading to safeguard against environmental variation, known as evolutionary bet-hedging (Beaumont et al. 2009; Levy et al. 2012; Sarhan and Kokko 2007; Seger 1987; Starrfelt and Kokko 2012; Stumpf et al. 2002; Venable 2007). In a stochastically varying environment, a species that maximises its mean reproductive rate, or mean fitness, is not necessarily the strongest, since this could coincide with increased sensitivity to fluctuations in the environment. A bet-hedger is defined as a strategy that has lower mean fitness than its rival, but is selected over the rival since it has reduced variation in its fitness, due to being less sensitive to these fluctuations. For example, consider a simple habitat that fluctuates between a short wet season and a long dry season. Mean fitness would be maximised by adapting to the dry season. However, such an adaptation may result in terrible performance during the wet season. A generalist, who is well-adapted to both seasons, will have lower mean fitness, but is protected from the environmental fluctuations and therefore has reduced variation in its fitness across seasons.

An ecological example of bet-hedging can be observed in the delayed germination strategy in desert annuals (Cohen 1966; Philippi 1993; Venable 2007). These plants release multiple seeds, most of whom germinate in the next season with a fraction remaining dormant until future seasons. Such a strategy reduces mean fitness, since some dormant seeds may be lost before germination. However, this strategy also reduces the variation in fitness, because it ensures that not all offspring will die if the next season is bad. Bet-hedging adaptations have also evolved in microbial communities under turbulent environments (Beaumont et al. 2009; Levy et al. 2012). These examples consider between-generational variation (Gillespie 1973), whereby all individuals experience the same conditions at any time. Mathematically, adaption to counter this type of variation is easily described and understood assuming evenly mixed populations of species (Gillespie 1973; Hopper 1999; Starrfelt and Kokko 2012).

Environmental variation can also act locally on individuals, causing within-generational (or demographic) variation. In this context, the fitness of an individual can be different from that of another individual of the same type at a given time, but both will have fitness drawn from the same distribution. One example is where predation levels across the habitat are variable. Assuming a cost of spreading offspring across numerous sites, the strategy to maximise fitness corresponds to choosing a single nesting location. However, since this site could be predated, a bet-hedger could evolve that spreads offspring across numerous sites to reduce the predation risk. An ecological example of potential bet-hedging against within-generational variation has been observed in female sierra dome spiders (Watson 1991). These females exhibit a multiple paternity strategy, whereby the primary mate is the victor of a fight among potential suitors, and secondary mates are selected at random. Mean fitness would be maximised by only selecting the primary mate, but random secondary mating hedges against the fight only taking place between weak suitors. Many other examples are similar and focus on multiple-paternity as a bet-hedging strategy (Fox and Rauter 2003; Sarhan and Kokko 2007; Watson 1991; Yasui 2001). Other work has identified strategies in Cabbage Butterflies (Root and Kareiva 1984) and Aphids (Ward and Dixon 1984) that potentially evolved as bet-hedgers against within-generational variation.

Despite the ecological observations, mathematical models in well-mixed populations lead to the conclusion that such variation does not drive evolutionary adaption unless the population is unrealistically small (Gillespie 1974; Hopper 1999; Hopper et al. 2003), contradicting and challenging the ecological observations (Courtney 1986; Hopper 1999; Hopper et al. 2003). Such challenges have potentially led to the lack of examples of bet-hedging against within-generational variation in recent literature, apart from cases restricting themselves to small population sizes (Sarhan and Kokko 2007).

Real populations are often not well-mixed and typically exist within some defined population structure, such as spatial or social structure. Some population structures consist of distinct groups of individuals within patches (or demes). Bet-hedging in such populations has been investigated using metapopulation models and deme-structured models (Lehmann and Balloux 2007; Shpak 2005; Shpak and Proulx 2007; Yasui and Garcia-Gonzalez 2016). These cases have demonstrated that within-generational bet-hedging strategies can evolve in metapopulations, provided the deme (or patch) contains sufficiently few individuals. Here we generalise the study of bet-hedging to graph structured populations, which can capture interaction/competition structure as well as deme-structure. The benefit of graph structure over metapopulations is that different types of contact structure can be considered. For example, populations where individuals interact and compete on a local scale but not within closed groups of individuals, such as competition in epithelial cells (Renton and Page 2019) and cancer growth (Hindersin et al. 2016). One distinction is that individuals do not necessarily interact with the neighbours of their neighbours, which can capture interactions such as social behaviour and spatially constrained competition. Mathematically, evolution in structured populations is described by evolutionary graph theory (Lieberman et al. 2005; Ohtsuki et al. 2006), which we build upon to incorporate variation. By analysing the evolutionary process in structured populations, we show that both between- and within-generational bet-hedging can be favoured in the evolutionary process, regardless of population size. This supports the conclusions from metapopulations in providing an explanation for the ecological observations. We also discuss how different types of structure impact selection for within-generational bet-hedging.

## Evolutionary model

To determine the impact of population structure on the evolution of bet-hedging strategies we model the dynamics of the process. We are interested in when a bet-hedging strategy has a competitive advantage over the non-bet-hedging strategy, which we call the normal-type. Evolution in structured populations (generally in a non-variable environment) is described by evolutionary graph theory (Antal et al. 2006; Broom et al. 2010; Broom and Rychtář 2008; Hindersin et al. 2016; Lieberman et al. 2005; Renton and Page 2019). Here, population structure is represented by an undirected connected graph in which connections between individuals represent potential for competition. This framework is an extension of the Moran process (Moran 1958) to structured populations. Metapopulation and deme-structured models fit this framework if we consider each patch (or deme) to be akin to a cluster of individuals who are connected, and the connections between patches to be represented by links between these clusters.

We consider a population with two types (or strategies) of individuals, the bet-hedging strategy *M* and the normal-type strategy *R*, either of which can play the role of the resident. The population structure is defined by a graph $G=(V,E)$, where *V* is the set of nodes and *E* is the set of edges between these nodes. The biological interpretation is that individuals in the population each occupy a node, with only one individual per node. The edges represent competition between individuals, in the sense that individuals can only place their offspring onto connected nodes.

**Fig. 1:**
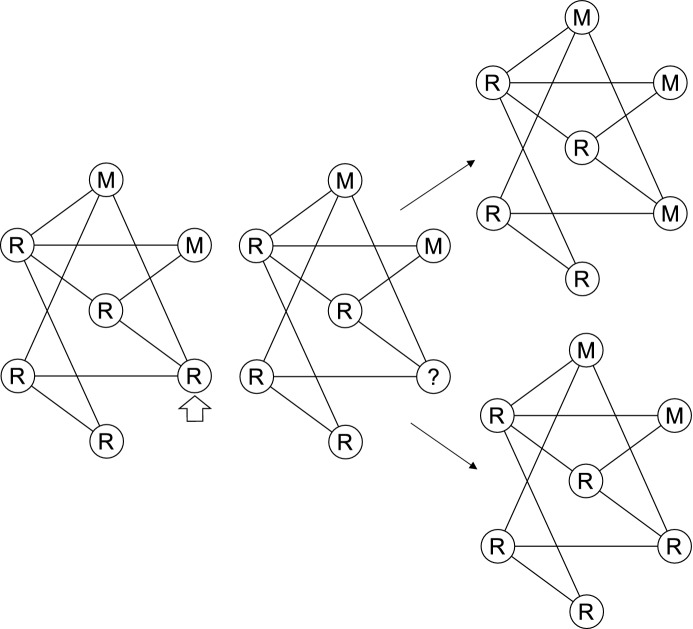
The update dynamics of the evolutionary process. Firstly, an individual is randomly selected for death, indicated by the white arrow (left-most image). This results in a vacant node in the population, which the neighbouring individuals can then compete to fill (middle image). One of these individuals is then selected for birth, with probabilities proportional to their fitness, and the vacant node updates to become the type of selected individual (right-most image). Either the selected node becomes a bet-hedger *M* (top-case) or a normal-type *R* (bottom-case)

Following Argasinski and Broom (2013), Roff (2008), and Wild and Taylor (2004), the fitness of an individual is proportional to its birth rate. Therefore, an individual is first selected to die at random, resulting in a vacant node in the population. The neighbouring (connected) individuals of this node then compete to replace with an identical offspring, with probability proportional to their fitness (Fig. 1). Since offspring are identical, there is no further mutation until one strategy eliminates the other. Following Ohtsuki et al. (2006), we refer to these dynamics as death-birth with selection on birth. Other dynamics have been proposed for evolution in structured populations, which we highlight in the discussion.

Traditional evolutionary graph theory dynamics do not capture variation in fitness, which is present in many real-world populations. To incorporate this we treat fitness as a random variable, the changes in which can be considered as changes in the local conditions. We assume that offspring inherit the fitness distribution of their parent, rather than the absolute fitness value. This general definition of fitness can account for either between and/or within-generational variation by changing correlations between each random variable. Many real-world examples (Beaumont et al. 2009; Gravenmier et al. 2018; Olofsson et al. 2009; Sarhan and Kokko 2007; Tufto 2015; Venable 2007; Yasui 2001; Yasui and Garcia-Gonzalez 2016; Yasui and Yoshimura 2018) can be modelled using random variables to capture the variability in future conditions. Modelling in this manner allows us to focus on the effect of variation. However, if applied to real populations, the explicit environmental changes may need to be modelled more directly.

**Fig. 2:**
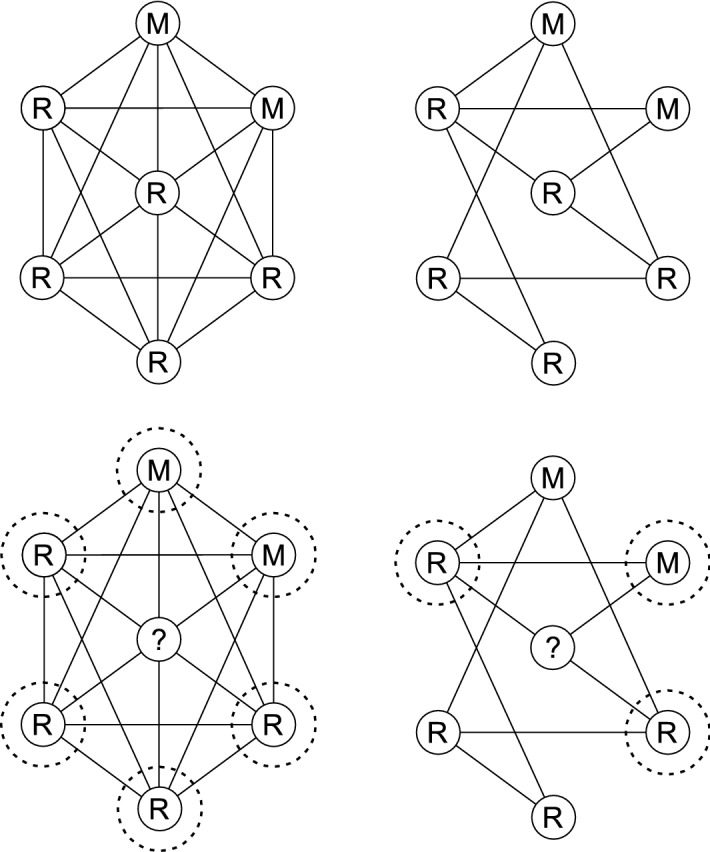
Figure showing how node-degree changes the selection group size. The two figures on the left show the selection group for a high degree focal node and the two figures on the right for a low degree focal node. The top figures indicate the graphs before an individual is selected for death, and the lower two figures show the resulting selection groups. After an individual is selected for death (central node), the connected individuals (in dashed circles) compete to replace this individual. We refer to these connected individuals as the selection group. The different structures can influence the number of nodes in the selection group, with a higher degree node having a much larger selection group

To describe how this stochastic process changes, we define the probability of moving from one state to another, where a state represents which nodes are occupied by *M* and which are occupied by *R*. From any state *S* that is not all *M* or all *R*, we can move to a state $S^+$ with more type *M* individuals (bet-hedgers) or $S^-$ with more type *R* individuals (normal-types). To move from *S* to $S^+$ we require a normal-type to die followed by selecting a bet-hedger for reproduction. For a given node *j*, the probability of death is 1/*N*. After *j* is selected for death, the neighbouring individuals, which we will refer to as the selection group (Fig. 2), compete to replace *j*. We will refer to the probability of selecting a certain type (given that an individual has been selected for death) as the selection probability of that type. Therefore, the probability of moving to a state $S^+$ is given by summing the products of the probability that each normal-type individual is selected for death multiplied by the corresponding bet-hedger selection probability. That is,

1 $$\begin{aligned} P(S \rightarrow S^+)= \sum \limits _{j=1}^N\frac{1}{N}R_j^SP(\text {type M selected to reproduce } | \text { j dies and state S}) \end{aligned}$$

where $R_j^S=1$ if the individual in node *j* is a normal-type in state *S*, and zero otherwise. Similarly, the probability of moving to a state with more *R* individuals is given by

2 $$\begin{aligned} P(S \rightarrow S^-)= \sum \limits _{j=1}^N\frac{1}{N}M_j^SP(\text {type R selected to reproduce } | \text { j dies and state S}), \end{aligned}$$

where $M_j^S=1$ if and only if the individual in node *j* is a bet-hedger in state *S*, and zero otherwise. These two probabilities dictate how the system evolves at each time step, and therefore provide a measure of the relative strength between the competing strategies.

## The effect of fitness variation on bet-hedger selection probability

An obvious measure of variation is the variance, which is useful since it is easily calculated. However, it has limitations; for example, if two distributions have equal mean and variance it gives no insight into which is more varied. This information is captured in the higher order moments of the distributions, such as the skew and kurtosis. A more comprehensive representation of variation is given by the convex order (Shaked and Shanthikumar 2007; Wilkinson and Sharkey 2018), such that if one distribution is greater than another in convex order then it is more variable. Convex order describes variability by ordering the expected values of convex functions, which are sensitive to the variation.

For two random variables *X* and *Y*, we say that *X* is less than *Y* in convex order (and therefore less variable than *Y*), denoted $X \le _{cx} Y$, if and only if ${\mathbb {E}}[\phi (X)] \le {\mathbb {E}}[\phi (Y)]$ for all convex functions $\phi$. A useful result that can be obtained from convex ordering is (Shaked and Shanthikumar 2007)

$$\begin{aligned} X \le _{cx} Y \implies {\mathbb {E}}[X] = {\mathbb {E}}[Y], \text {Var}(X) \le \text {Var}(Y), \end{aligned}$$

so if one random variable is less than another in convex order then its variance cannot be larger than the other. Establishing convex order can be difficult, but there are methods for doing this and under certain circumstances, this ordering of distributions reduces to ordering of the variance of the distributions (Shaked and Shanthikumar 2007). However, for our purposes, we only need to use this as a precise ordering of variability between any two distributions.

In the evolutionary process, selective pressure is governed by the selection probabilities on the right-hand side of Eqs. (1) and (2). For any given replacement event, the selection group consists of *m* bet-hedgers and *n* normal-types, so the selection probability depends on *m* and *n*. The bet-hedger selection probability can be shown (“Appendix” A) to reduce to

3 $$\begin{aligned} P(M \text { reproduces }| m \text { type } M \text { and } n \text { type } R)&={\mathbb {E}}\left[ \frac{\sum \limits _{i=1}^mf_i^M}{\sum \limits _{i=1}^mf_i^M+\sum \limits _{j=1}^{n}f_j^R} \right] , \end{aligned}$$

where $f_i^M$ is the fitness of a bet-hedger *i* and $f_j^R$ is the fitness of a normal-type *j*. Here, the bet-hedgers in the selection group (immediate neighbours of the individual selected for death) are labelled from 1 to *m* and the normal-types are labelled from 1 to *n*, so that $m+n=k$ where *k* is the size of the selection group. Noting that the selection probability of a normal type and the selection probability of a bet hedger sum to 1, the strength of selection can be represented solely by the bet-hedger selection probability. Equation (3) is the expected value of a convex function of normal-type fitness. Therefore, by the definition of convex order, increasing the variation of normal-type fitness in convex order can only increase the selection probability of the bet-hedger.

The bet-hedger selection probability can be also written as 1 minus the normal-type selection probability, which gives

$$\begin{aligned} P(M \text { reproduces }| m \text { type } M \text { and } n \text { type } R)&=1 - {\mathbb {E}}\left[ \frac{\sum \limits _{j=1}^nf_j^R}{\sum \limits _{i=1}^mf_i^M+\sum \limits _{j=1}^{n}f_j^R} \right] . \end{aligned}$$

The second term here is a convex function of the bet-hedger fitness, which implies that decreasing the bet-hedger fitness variation through convex order can only decrease the value of this term, thus increasing the bet-hedger selection probability. Therefore, bet-hedger performance increases through either the environment experienced by normal-type individuals becoming more variable or by reducing its own fitness variability.

To obtain the selection probability (Eq. (3)), the bet-hedger fitness is averaged over the total fitness of the surrounding individuals (“Appendix” A). Assuming that the proportion of bet-hedgers to normal-types remains approximately constant $(m/n \approx \gamma )$, the selection probability can be transformed to depend on sample averages,

$$\begin{aligned} P(M \text { reproduces }| m \text { type } M \text { and } n \text { type } R)&={\mathbb {E}}\left[ \frac{\gamma (\sum \limits _{i=1}^mf_i^M)/m}{\gamma (\sum \limits _{i=1}^mf_i^M)/m+(\sum \limits _{j=1}^{n}f_j^R)/n} \right] . \end{aligned}$$

For within-generational variation, each individual can sample a different value at the same time. Therefore, as the selection group size increases, these sample averages become less sensitive to normal-type variation, due to the law of large numbers. Consequently, the selection probability becomes less sensitive to this variation as we increase selection group size, and for large selection groups, selection for reduced within-generational variation is diminished. This explains why in large well-mixed populations, within-generational bet-hedging should not evolve (Gillespie 1974). However, since the selection group depends on the degree of the node chosen for death (see Fig. 2 for an illustration), if the degree of this node is low then within-generational variation can have a large impact on the selection probability, regardless of population size. By taking small clusters of fully connected individuals, inter-connected with a sparse number of edges, we can create metapopulation-like graphs. In this case, if the cluster size is small, the selection group will be small and within-generational bet-hedging can evolve, agreeing with predictions from metapopulation models (Lehmann and Balloux 2007; Shpak 2005; Shpak and Proulx 2007; Yasui and Garcia-Gonzalez 2016).

For between-generational variation, individuals experience identical conditions during a generation. This is modelled by having all individuals of a given type sample the same value. Therefore, the selection probability can be written as

$$\begin{aligned} P(M \text { reproduces }| m \text { type } M \text { and } n \text { type } R)&={\mathbb {E}}\left[ \frac{mf_1^M}{mf_1^M+nf_1^R} \right] . \end{aligned}$$

Assuming the proportion of bet-hedgers to normal-types is constant ($m/n \approx \gamma$), this becomes

$$\begin{aligned} P(M \text { reproduces }| m \text { type } M \text { and } n \text { type } R)&={\mathbb {E}}\left[ \frac{\gamma f_1^M}{\gamma f_1^M+f_1^R} \right] , \end{aligned}$$

for any selection group size. Therefore, for between-generational bet-hedging there is no diminishing effect due to large selection groups since there is no dependence on selection group size, and between-generational variation will have a large impact for all graphs and population sizes. Since there is no significant impact of population structure on selection, and evolution of between-generational bet-hedging has been widely explored mathematically (Gillespie 1974; Hopper 1999; Starrfelt and Kokko 2012), we focus on within-generational bet-hedging for the remainder of this paper, and hence will drop the within-generational prefix in what follows.

### Approximate result for variance

For evolutionary bet-hedging models, approximate results are often derived using a second order Taylor approximation (Frank and Slatkin 1990; Gillespie 1974; Rice 2008; Rice and Papadopoulos 2009; Shpak 2005; Shpak and Proulx 2007; Starrfelt and Kokko 2012). Following this approach, we apply a two-dimensional second-order Taylor approximation about the mean fitness of both types, which yields (“Appendix”  B.1)

4 $$\begin{aligned}&P(M \text { reproduces }| \ m \text { type } M \text { and } n \text { type } R) \nonumber \\&\quad \approx \frac{m\mu _M}{(m\mu _M+n\mu _R)}+\frac{mn\mu _M}{(m\mu _M+n\mu _R)^3}\sigma ^2_R\nonumber \\&\qquad -\frac{m}{(m\mu _M+n\mu _R)^2}\sigma ^2_M+\frac{m^2\mu _M}{(m\mu _M+n\mu _R)^3}\sigma ^2_M \end{aligned}$$

where $\mu _Z$ and $\sigma ^2_Z$ are the expected value and the variance of the type $Z \in \{M,R\}$ fitness distribution, respectively. This suggests that mean and variance are key parameters controlling the selection probability.

The coefficient of $\sigma _R^2$ is positive, so increasing the variation of the normal-type fitness through the variance is likely to increase the bet-hedger selection probability. The total coefficient of $\sigma ^2_M$ is negative, so decreasing the bet-hedger variation through the variance is also likely to increase the bet-hedger selection probability. We again observe that selection for bet-hedging depends on the size of the selection group rather than population size, since the variance dependent terms in Eq. (4) diminish with *m* and *n*. The diminishing effect occurs since the order and *m* and *n* in the denominator are higher than in the numerator.

For the bet-hedger to be favoured in a given replacement event, we require the selection probability of the bet-hedger in this event to be larger than the selection probability of the normal-type in the opposite event. That is, we require the bet-hedger selection probability from *m* bet-hedgers against *n* normal-types to be larger than the normal-type selection probability from *m* normal-types against *n* bet-hedgers. Treating the bet-hedger variance as fixed, the normal-type variance at which these are equal, which we call the critical normal-type variance, is approximated by (“Appendix” B.2)

5 $$\begin{aligned}&\sigma _R^2({k,m})\nonumber \\&\quad \approx \frac{1}{\Big ((k^2 - 3km + 3m^2)\mu _R^2 - \mu _M(k^2 - 6km + 6m^2)\mu _R + \mu _M^2(k^2 - 3km + 3m^2)\Big )k\mu _M} \nonumber \\&\qquad \times \Bigg [\mu _R^5m^2(k - m)^2 + 2(k - m)\mu _M\big (\frac{5}{2}m^2 - \frac{5}{2}km + k^2\big )m\mu _R^4 \nonumber \\&\qquad + \Big ((k^4 - 6k^3m + 16k^2m^2 - 20km^3 + 10m^4)\mu _M^2 + k\sigma ^2_M(k^2 - 3km + 3m^2)\Big )\mu _R^3 \nonumber \\&\qquad - \Big ((k^4 - 6k^3m + 16k^2m^2 - 20km^3 + 10m^4)\mu _M^2 + k\sigma ^2_M(k^2 - 6km + 6m^2)\Big )\mu _M\mu _R^2 \nonumber \\&\qquad - 2\mu _M^2\Big ((k - m)\big (\frac{5}{2}m^2 - \frac{5}{2}km + k^2\big )m\mu _M^2 - k\sigma ^2_M\frac{(k^2 - 3km + 3m^2)}{2}\Big ) \mu _R - m^2(k - m)^2\mu _M^5\Bigg ]. \end{aligned}$$

Alternatively, we can treat normal-type variance as fixed and find the critical bet-hedger variance by rearranging Eq. (5) to find $\sigma ^2_M$ (not shown). Taking the derivative of Eq. (5) with respect to *m* we obtain

$$\begin{aligned}&\frac{\partial {\sigma _R^2({k,m})}}{\partial m} \\&\quad = \frac{1}{\mu _M((k^2 - 3km + 3m^2)\mu _R^2 - \mu _M(k^2 - 6km + 6m^2)\mu _R + \mu _M^2(k^2 - 3km + 3m^2))^2k} \\&\qquad \times \Bigg [-2\Big ((k^2 - \frac{3}{2}km + \frac{3}{2}m^2)\mu _M^2 + \mu _R(k^2 + 6km - 6m^2)\frac{\mu _M}{2} \\&\qquad + \mu _R^2(k^2 - \frac{3}{2}km + \frac{3}{2}m^2)\Big ) \\&\qquad \times \big ((k - m)\mu _M + \mu _Rm\big )(k - 2m)\big (m\mu _M + \mu _R(k - m)\big )(\mu _M - \mu _R)^3\Bigg ]. \end{aligned}$$

This can be written as

$$\begin{aligned} f(m)=\frac{\partial {\sigma _R^2({k,m})}}{\partial m}=\frac{g(m)h(m)}{z(m)}, \end{aligned}$$

where

$$\begin{aligned} g(m)=&2(k^2 - 3/2km + 3/2m^2)\mu _M^2 + \mu _R(k^2 + 6km - 6m^2)\mu _M \\&+ 2\mu _R^2(k^2 - 3/2km + 3/2m^2), \\ h(m)=&\Big ((k - m)\mu _M + \mu _Rm \Big )(k - 2m)\Big (m\mu _M + \mu _R(k - m)\Big ), \\ z(m)=&\mu _M\Big ((k^2 - 3km + 3m^2)\mu _R^2 - \mu _M(k^2 - 6km + 6m^2)\mu _R\\&+ \mu _M^2(k^2 - 3km + 3m^2)\Big )^2k . \end{aligned}$$

Both *h*(*m*) and *z*(*m*) are strictly positive for $m \le k/2$. Therefore, we only need to consider the sign of *g*(*m*). The derivative of *g*(*m*) with respect to *m* is a negative function of *m*, so the minimum value occurs at $m=k/2$. Since $g(k/2)>0$, *g*(*m*) must be positive for all $m \le k/2$. Since all three functions are positive over the range of *m* that we consider, *f*(*m*) is positive for all $m \le k/2$. Therefore, the critical normal-type variance is an increasing function of *m*, and the maximum critical normal-type variance occurs in the evenly-mixed scenario, $m=n=k/2$, where

6 $$\begin{aligned} \sigma _R^2({k,k/2}) \approx \frac{(\mu _R^2-\mu _M^2)(\mu _M+\mu _R)k + 4\mu _R\sigma ^2_M}{4\mu _M}. \end{aligned}$$

In this scenario, the critical normal-type variance grows linearly with the selection group size *k*, showing that selection for within generational bet-hedging quickly diminishes with selection group size. In the case where $\sigma ^2_M = 0$, the critical normal-type variance is proportional to *k*, so any increase in *k* requires a increase in the critical normal-type variance. In the general case, if both *k* and $\sigma _M^2$ increase by the same proportion, then the critical normal-type variance also increases by this proportion.

### Implications for the fixation probability

The results in Sects. 3 and 3.1 focus on the relative strength of each strategy for a given selection event. However, the evolutionary process consists of multiple selection events with different selection groups. Therefore, the fixation probability determines the overall strength of each strategy. This is the probability that an initial subset of mutants takes over the population, and is a popular measure to compare different strategies within evolutionary theory (Altrock and Traulsen 2009; Broom and Rychtář 2008; Czuppon and Traulsen 2018; Giaimo et al. 2018; Lieberman et al. 2005; Traulsen and Hauert 2010). Since increasing variation in normal-type fitness (or decreasing bet-hedger fitness variation) increases the relative strength of bet-hedgers, we assume that bet-hedger fixation probability will also increase.

Assuming that bet-hedger fixation probability is increasing with normal-type variation, we want to find the level of variation above which the bet-hedger becomes favoured in the evolutionary process; i.e. the fixation probability of a bet-hedger invading a normal-type population is higher than the normal-type invading bet-hedgers. We call this the overall critical normal-type variation. Subject to the assumptions of the Taylor approximation, we showed that there is a level of variation in the normal-type fitness above which the bet-hedger is favoured for a given selection event. We assume that there is also a critical normal-type variation for arbitrary fitness distributions (where the assumptions of the Taylor approximation may not be satisfied). For certain distributions, such as the gamma distribution, this can be calculated using numerical methods (e.g. “Appendix” C.1). Alternatively, this critical normal-type variation can be approximated by setting the variance of the distribution to be given by Eq. (5). Since there is a level of variation above which, for a given selection group, the bet-hedger will be favoured, eventually the bet-hedger will be favoured in every selection group. Therefore, the overall critical normal-type variation must exist.

On *k*-regular graphs, the conclusions from the selection probability can easily be applied to fixation probability, since each selection group is of size *k*. Here, if the bet-hedger is favoured in every scenario for a size *k* selection group, they must be favoured overall. From the Taylor approximation, if the bet-hedger is favoured in the evenly-mixed scenario then the bet-hedger is favoured in every scenario, so we assume that the critical normal-type variation for the evenly-mixed scenario provides an upper bound for the overall critical normal-type variation. Extending the conclusions to arbitrary graphs is less clear, since there can be variability in node-degree. In such cases, it is not obvious how the cumulative effects of different selection group sizes will affect the relative strength of bet-hedgers (the upper bound can be determined by looking at the maximum selection group size on the network, but this may be a very loose upper bound). To investigate this, and to confirm that our assumptions hold, we now investigate the fixation probability numerically.

## Numerical results

Here we investigate the fixation probability numerically using stochastic simulations, in order to test the assumptions from Sect. 3.2. For a fitness distribution, we require a distribution bounded below by zero. Right-skewed distributions are common in many biological systems, so we choose to use gamma distributions to represent fitness for both bet-hedgers and normal-types. We opt to use gamma over other right-skewed distributions since for the gamma distribution, convex order reduces to ordering the variance, which enables the variation of the distribution to be easily controlled.

### Regular graphs

**Fig. 3:**
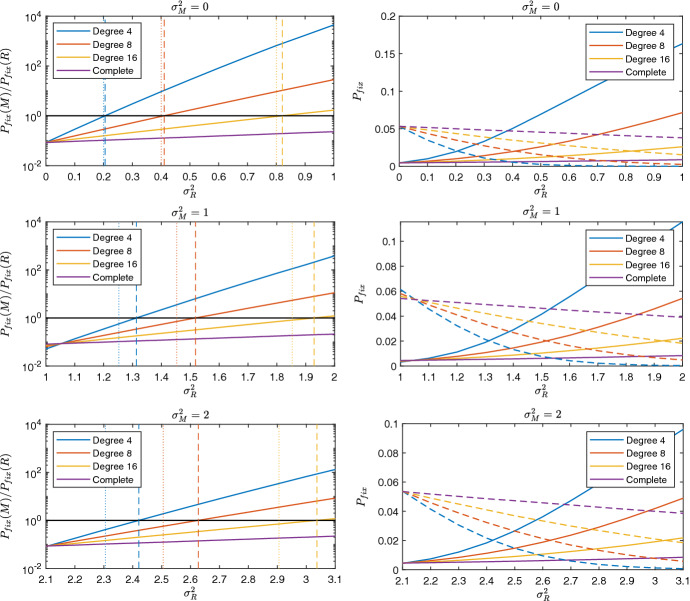
The impact of within-generational variation on the overall relative strength of bet-hedgers on four 50 node *k*-regular graphs. Bet-hedger has mean fitness equal to 0.95 with normal-type mean fitness equal to 1. The left-hand figures show the ratio of bet-hedger to normal-type fixation probability, calculated from a single mutant of the invading type. The right-hand figures show the fixation probabilities, with the solid curves indicating the bet-hedger and the dashed-curves indicating the normal-type. Along the x-axis we change the variance of the normal-type fitness distribution, $\sigma _R^2$. Moving from the top through the middle to the bottom subgraphs, the variance of the bet-hedger fitness distribution, $\sigma _M^2$, increases. When variance is non-zero, we assume fitness is given by a gamma distribution (the parameters of which are uniquely determined by the mean and variance), so changing the variance changes the convex order (and therefore changes the variation in the distribution). On the left-hand figures, the black horizontal line at 1 is used to indicate which strategy is favoured. If the ratio is below 1 the normal-type is favoured, and above 1 the bet-hedger if favoured. The overall critical normal-type variance is the *x*-coordinate when each ratio crosses 1. The upper bounds are indicated by the dashed vertical lines and the approximate upper bounds are marked by the dotted vertical lines

We consider four 50 node graphs: a complete graph, and three *k*-regular random graphs, with degrees 16, 8 and 4. The relative strengths of the normal-type and the bet-hedger is determined by the ratio of their fixation probabilities. The overall critical normal-type variation for each graph is given by the variation at which this ratio is equal to 1. We compare this to the upper bounds predicted by the evenly-mixed critical normal-type variation, calculated numerically (see “Appendix” C.1) and using the Taylor approximation [Eq. (6)].

Figure 3 illustrates that increasing the normal-type variation increases the bet-hedger fixation probability and decreases the normal-type fixation probability for all graphs, as expected based on the assumptions in Sect. 3.2. Since the selection probability is very sensitive to the degree of the graph [Eq. (3)], for graphs with high degree, such as the complete graph, variation has little impact on the selection probability. This is reflected in the slight impact of normal-type variance on the fixation probability and is consistent with established results in well-mixed populations (Gillespie 1974; Seger 1987). For graphs with low average degree, variation has a significant effect on the fixation probability and can be a key factor in the evolutionary process. As we increase the variation in bet-hedger fitness (measured through increasing the variance, $\sigma _M^2$) we observe the same pattern (Fig. 3), however the critical normal-type variation required for the bet-hedger to be favoured increases. This is in line with the results from the Taylor approximation (Eq. 5), which suggested that critical normal-type variation is an increasing function of bet-hedger variation.

We argued in Sect. 3.2 that the evenly-mixed critical normal-type variation provides an upper bound on the overall critical normal-type variation. This upper bound in fact provides a good approximation to the overall critical normal-type variation for each graph tested (the dashed lines in Fig. 3 are at a slightly higher normal-type variance than where the ratio crosses 1), which we can use to gain insight into how much variation is required for the bet-hedger to be favoured in regular graphs. Under the gamma distribution, the evenly-mixed critical normal-type variation linearly increases with selection group size when the bet-hedger has no variation in their fitness (“Appendix” C.2), showing that increasing selection group size can quickly suppress selection for bet-hedging strategies. Comparing the Taylor approximation to the computed upper bound (the dotted and dashed lines in Fig. 3, respectively), we observe that this provides a rough approximation to the upper bound when the variance required for the bet-hedger to be favoured is low. However, as the variance required increases (in this case by increasing selection group size or increasing the bet-hedger variation) the discrepancy between the two increases. This is because as the normal-type fitness distribution becomes more variable, the higher order moments increase, which causes the assumptions underpinning the Taylor approximation to no longer hold.

### Impact of degree heterogeneity

On *k*-regular graphs, we have shown that the average degree controls selection for bet-hedging. Such graphs have no variability in the degree of different nodes, however, in real populations we would expect some level of degree variability. Here, we consider the effect of degree variability on selection for bet-hedging.

We first consider the star graph and the circle, which have the same average degree in the limit of large population size but different degree variability. On the star graph, there is one focal individual who is connected to every other individual. All other individuals are only connected to this focal individual. This graph has high degree variability, with one node having degree $N-1$ and all others having degree 1, where *N* is the population size. On the circle, individuals are connected in a loop, so all nodes have degree 2 and there is no variability.

For the star graph, there are a few possible distinct transitions that can occur. If a bet-hedger is in the central node, the changes that can happen to the system are either this node dying and being replaced by a normal-type from the leaf nodes, or a normal-type leaf node dying and being replaced by a bet-hedger from the central node. Similarly, if the central node is a normal-type, either this node can die and be replaced by a bet-hedger from a leaf node, or a bet-hedger leaf node can die and be replaced by a normal-type from the central node. Due to the symmetry of the leaf nodes, we can group all these events together so that we only have these four possible state transitions to consider. Let $p_{i,i+1}^{MM}$ denote the probability that the system transitions from a state with a bet-hedger in the central node and *i* bet-hedgers on the leaves to a state with a bet-hedger on the central node and $i+1$ bet-hedgers on the leaves. Let $p_{i,i}^{MR}$ denote the probability that the system transitions from a state with a bet-hedger in the central node and *i* bet-hedgers on the leaves to a state with a normal-type in the central node and *i* bet-hedgers on the leaves. From Eqs. (1) and (2), these transition probabilities are given by

7 $$\begin{aligned} p_{i,i+1}^{MM}=&\frac{N-1-i}{N},\nonumber \\ p_{i,i}^{MR}=&\frac{1}{N}{\mathbb {E}}\left[ \frac{\sum \limits _{j=1}^{N-1-i}f^R_j}{\sum \limits _{j=1}^{i}f^M_j+\sum \limits _{j=1}^{N-1-i}f^R_j}\right] , \end{aligned}$$

8 $$\begin{aligned} p_{i,i-1}^{RR}=&\frac{i}{N},\nonumber \\ p_{i,i}^{RM}=&\frac{1}{N}{\mathbb {E}}\left[ \frac{\sum \limits _{j=1}^{i}f^M_j}{\sum \limits _{j=1}^{i}f^M_j+\sum \limits _{j=1}^{N-1-i}f^R_j}\right] . \end{aligned}$$

Selection is only taking place when an individual on a leaf node replaces the central node (Eqs. (7) and (8)). This is because when a leaf node dies only the central node can replace this, so there is no competition-based selection taking place. When the central node is replaced, the selection group size is equal to the number of leaves, which is $N-1$ for population size *N*. Therefore, for a sufficiently large population, the impact of within-generational variation on the selection probability rapidly diminishes, resulting in no selection for within-generational bet-hedging on a large star graph.

For the circle, suppose there is a single cluster of connected bet-hedger individuals with no normal-type individuals between them. The symmetry of the graph allows us to consider the number of bet-hedger individuals rather than their locations, since the group of bet-hedgers can only change at the two boundaries where they meet normal-types (Broom et al. 2010). Denoting the probability of moving from a state with *i* bet-hedgers to $i+1$ bet-hedgers by $p_{i,i+1}$, and the reverse process by $p_{i,i-1}$, we can describe the transition probabilities between different states with the following equations

$$\begin{aligned} p_{1,0}=&\frac{1}{N},\\ p_{i,i+1}=&\frac{2}{N}{\mathbb {E}}\left[ \frac{f^M_j}{f^M_j+f^R_j}\right] , i<N-1\\ p_{i,i-1}=&\frac{2}{N}{\mathbb {E}}\left[ \frac{f^R_j}{f^M_j+f^R_j}\right] , i>1\\ p_{N-1,N}=&\frac{1}{N}, \end{aligned}$$

where *j* is arbitrary since the fitness distributions for a given type are independent and identically distributed. From this we can see that on the circle within-generational variation will always have an influence on selection, since there is no dependence on *N*, potentially paving the way for a bet-hedging strategy to evolve. Although an extreme case, this shows that increasing the variability in the degree distribution of the graph (by going from no variation on the circle to high variation on the star) may reduce the selection for bet-hedging.

**Fig. 4:**
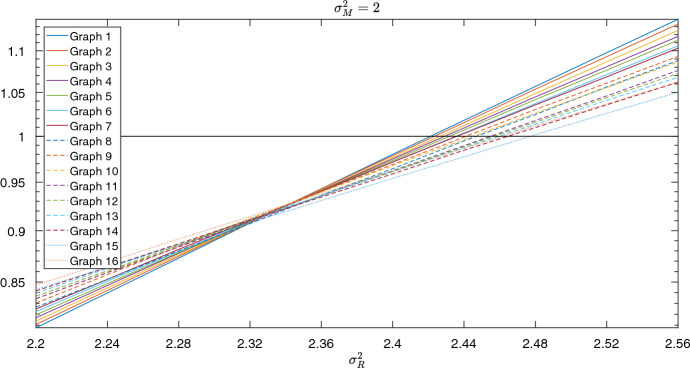
The impact of variance in the degree distribution on the overall critical normal-type variation. We consider sixteen 8 node graphs with average degree equal to 4, each with a different variance in their node degree distribution. Along the x-axis we change the variance of the normal-type fitness distribution, $\sigma _R^2$. The y-axis shows the ratio between the bet-hedger fixation probability and normal-type fixation probability

To investigate this further, we generate a sample of graphs with fixed population size and average node degree but different degree variability (measured using the variance of the degree distribution). Generating 1,000,000 random graphs (Erdős and Rényi 1960) with $N=8$ and average degree equal to 4 results in 16 unique degree distribution variances being sampled. From these graphs, we select a cohort of 16 graphs by randomly selecting a single graph for each variance. We label these numerically from “Graph 1” to “Graph 16”, where increasing the index corresponds to an increase in the degree distribution variance.

For each graph, we assume the bet-hedger has gamma distributed fitness with mean 0.95 and variance 2. Normal-type fitness is gamma distributed with mean 1, and we change the variation to numerically determine the overall critical normal-type variation. For each value of normal-type variation, we perform 30,000,000 stochastic simulations to identify the fixation probability of a single mutant of each type in a resident population of the other, from which we calculate the ratio of fixation probabilities, with the results shown in Fig. 4. Using this, we determine the critical normal-type variation, which gives the ordering: 1, 2, 3, 4, 5, 6, 7, 9, 10, 8, 11, 12, 13, 16, 14, 15. In the majority of cases, increasing variance in the degree distribution increases the overall critical normal-type variation, and therefore decreases selection for bet-hedging. There are however some outliers in this pattern (graphs 8 and 16 in this example). This is a common occurrence in evolutionary graph theory, where it has been found that under the death-birth with selection on birth dynamics considered here, although most random graphs suppress fixation probability relative to a well-mixed population, there are a small minority of outliers for which this is reversed (Hindersin and Traulsen 2015).

### Population size

We have shown that, for regular graphs, increasing the average degree of the graph increases the critical normal-type variation required for the bet-hedger to be favoured. From Sects. 3 and 3.1, the critical normal-type variation on such graphs should be independent of total population size. Here, we investigate the impact of population size on regular graphs using three populations sizes: $N=50$, $N=100$, $N=200$.

**Fig. 5:**
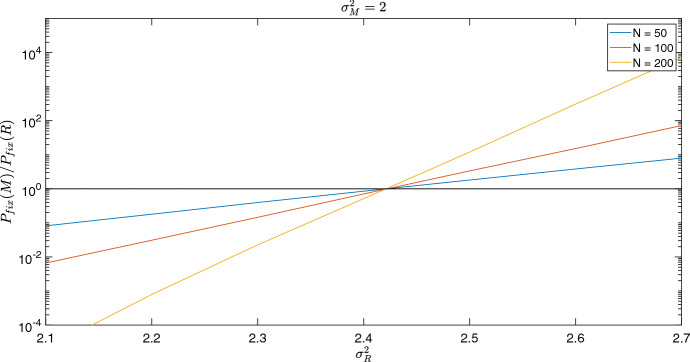
The impact of population size on the overall critical normal-type variation. We consider three *k*-regular random graphs with degree equal to 4 and population sizes: 50, 100 and 200. We change the normal-type variance, $\sigma _R^2$, along the x-axis. The y-axis shows the ratio between the bet-hedger fixation probability and normal-type fixation probability

For each population size, we assume that bet-hedgers have gamma distributed fitness with mean 0.95 and variance 2, and that normal-type have gamma distributed fitness with mean 1. To determine the overall critical normal-type variation, we vary the normal-type variation. We perform 1,000,000 stochastic simulations for each value of normal-type variance, first with the bet-hedger invading the normal-type and then with the normal-type invading the bet-hedger, from which we calculate the ratio of bet-hedger to normal-type fixation probabilities. Figure 5 shows that the population size does not have an influence on the critical normal-type variation, which is instead governed by selection group size. We see that larger population sizes amplify selection for the favoured type. This occurs since in a larger population, a rare invader is less likely to be randomly selected for death. Therefore, if the invader has a selective advantage over the resident, it will be more likely to have a chance to reproduce and gain a foothold than it would in smaller populations.

## Discussion

Evolutionary bet-hedging refers to the theory that the evolutionary process is sensitive to variation in fitness, with some species potentially accepting a decrease in their mean fitness to reduce this variation. A key area of discussion within evolutionary bet-hedging is the existence of strategies that potentially bet-hedge against within-generational variation; i.e. variations that affect individuals of the same type differently within each generation. Such strategies have been observed (Fox and Rauter 2003; Root and Kareiva 1984; Sarhan and Kokko 2007; Ward and Dixon 1984; Watson 1991; Yasui 2001), however mathematical theory has widely challenged their existence, instead suggesting that evolution should not select for this type of variation.

Traditional work has been mostly limited to well-mixed populations. Real populations however often exhibit some degree of structure, and this structure can have a significant impact on the evolutionary process. These impacts include amplifying the probability of advantageous mutants taking over a population (Lieberman et al. 2005) and facilitating the evolution of cooperative strategies in social dilemmas (Ohtsuki et al. 2006). Using models with metapopulation structure has demonstrated that within-generational variation can be important when the patches are sufficiently small (Lehmann and Balloux 2007; Shpak 2005; Shpak and Proulx 2007; Yasui and Garcia-Gonzalez 2016). By analysing bet-hedging strategies in graph structured populations, we have shown that within-generational variation can be a key factor in selection, and strategies that bet-hedge against such variation can be favoured in the evolutionary process, regardless of population size. In particular, provided that the average degree of the graph is reasonably low and degree variability is not too high, selection for within-generational bet-hedging is strong.

In such populations, bet-hedging strategies are likely to evolve, underpinning the results of some ecologists who have used bet-hedging against within-generational variation to explain observed strategies (Root and Kareiva 1984; Sarhan and Kokko 2007; Ward and Dixon 1984; Watson 1991). Many real-world population structures will have these properties, since individuals compete with a subset of the whole population and there will not be wide variability in the size of such competition groups. For example, the spread of cancer has been modelled using evolutionary graph theory frameworks (Hindersin et al. 2016). Recent results have shown the potential for between-generational bet-hedging within cancer cells (Gravenmier et al. 2018). Modelling the variable environment experienced by cancer cells with an evolutionary graph theory competition framework could provide evidence for the potential of within-generational bet-hedging in cancer cells. This scenario would not be directly evidenced by a subdivided population model since there are no clear divisions in the population, only local competition between the cells.

This paper has focused on death-birth with selection on birth dynamics. However, other evolutionary dynamics have been suggested for evolution in structured populations, such as birth-death with selection on death (Antal et al. 2006), where first an individual is randomly selected to reproduce, with the offspring replacing a neighbour selected with probability proportional to the inverse of their fitness. Under these dynamics, if fitness is taken to be a measure of survivability rather than birth rate, then adding population structure also allows bet-hedging against within-generational variation to take place. This is because here the selection groups in Eq. (3) only depend on local competition between the immediate neighbours. There are also dynamics that have global rather than local competition, such as birth-death with selection on birth (Lieberman et al. 2005) (where first an individual is selected to reproduce proportional to their fitness, with the offspring replacing a randomly selected neighbour) and death-birth with selection on death (Masuda 2009) (where first an individual is selected for death inversely proportional to their fitness, and is then replaced by a randomly selected neighbour). Within such global update mechanisms, evolution is unlikely to select for bet-hedging against within-generational variation. This is because global competition results in the selection probabilities always involving every individual within the population, so the effect is diminished by the law of large numbers. Within-generational bet-hedging is facilitated by local competition between subsets of the population.

The evolutionary graph theory framework is quite restrictive, in that it considers asexual reproduction, and many of the bet-hedging examples concern sexual reproduction. However, since our results only depend on the local competition aspect of the dynamics, it is reasonable to extend our conclusions to real-world evolutionary processes in which competition happens between small subsets of the population at any given time, going beyond the restrictions of the evolutionary graph theory framework. For example, in the multiple paternity scenario, although there is interaction between males and females, and males are competing to mate, once offspring are produced, the success of the offspring can be interpreted as competition between distinct females. In such a scenario, our result requires that the competition between these females is local, which is likely to be satisfied since an individual will not interact with all other individuals within their environment. This presented analysis focused on fixed population structures, whereas in real populations individuals may change who they interact with. Since our results only depend on the interaction structure at the time of the event, if the properties of the population structure do not significantly change over time our conclusions will still hold. Using evolutionary graph theory structure therefore provides further evidence that within-generational variation is important in empirical systems and within-generational bet-hedging is likely to be observed. This provides a theoretical basis for the existing observations and can motivate further empirical research to identify within-generational bet-hedging species, which has not been fully explored, perhaps due to the existing theoretical conclusions from well-mixed populations.

### Supplementary Information

Below is the link to the electronic supplementary material.

Supplementary material 1 (m 1 KB)

Supplementary material 2 (m 1 KB)

Supplementary material 3 (m 9 KB)

Supplementary material 4 (m 12 KB)

## Appendix A Selection probability

Given that an individual has been selected for death, one of the neighbours of this individual must be selected for birth, with probability one. We are therefore interested in deriving the probability that the selected individual is a bet-hedging type. We can assume without loss of generality that there are *m* bet-hedgers and *n* normal-type neighbours around the focal individual. We can label the bet-hedgers arbitrarily from 1 to *m* and the normal-types arbitrarily from 1 to *n*, such that bet-hedger *i* has fitness distribution $f_i^M$ and normal-type *j* has fitness distribution $f_j^R$. The individuals in the neighbourhood compete and a random individual is selected, with probability proportional to their fitness, to reproduce.

Once we have sampled the fitness values for each individual, the total fitness is given by $\sum \limits _{i=1}^mf_i^M+\sum \limits _{j=1}^nf_j^R$, and therefore the probability of selecting bet-hedger *i* is $f_i^M/(\sum \nolimits _{i=1}^mf_i^M+\sum \limits _{j=1}^nf_j^R)$, and normal-type *j* is $f_j^R/(\sum \nolimits _{i=1}^mf_i^M+\sum \limits _{j=1}^nf_j^R)$. Therefore, the probability of selecting any bet-hedger is given by

9 $$\begin{aligned} P(M \text { reproduces }| m \text { type } M \text { and } n \text { type } R)= \frac{\sum \limits _{i=1}^mf_i^M}{\sum \limits _{i=1}^mf_i^M+\sum \limits _{j=1}^nf_j^R}. \end{aligned}$$

When selecting which individual reproduces, we draw a random number from the uniform distribution between 0 and 1. If this number is smaller than Eq. (9) then we select a bet-hedger, otherwise we select a normal-type. Therefore, the selection probability of a bet-hedger, before sampling any of the fitness values, is given by

$$\begin{aligned} P(M \text { reproduces }| m \text { type } M \text { and } n \text { type } R)=P\left( Z<\frac{\sum \limits _{i=1}^mf_i^M}{\sum \limits _{i=1}^mf_i^M+\sum \limits _{j=1}^nf_j^R}\right) , \end{aligned}$$

where $Z\sim U(0,1)$. Defining $Y=\sum \nolimits _{j=1}^nf_j^R$ and $X=\sum \nolimits _{i=1}^mf_i^M$, we have

$$\begin{aligned} P\left( Z<\frac{X}{X+Y}\right)= & {} \int \limits _0^\infty \int \limits _0^\infty \int \limits _0^\frac{x}{x+y} f_{Z,X,Y}(z,x,y)dzdxdy \\= & {} \int \limits _0^\infty \int \limits _0^\infty \left( \int \limits _0^\frac{x}{x+y} f_{Z|X,Y}(z|x,y)dZ\right) f_{X,Y}(x,y)dxdy \\= & {} \int \limits _0^\infty \int \limits _0^\infty \left( \frac{x}{x+y}\right) f_{X,Y}(x,y)dxdy \\= & {} {\mathbb {E}}\left[ \frac{X}{X+Y}\right] , \end{aligned}$$

where $f_{Z,X,Y}(z,x,y)$ is the joint distribution function of *Z*,*X*, and *Y*, $f_{Z|X,Y}(z|x,y)$ is the conditional distribution function of *Z* given *X* and *Y*, and $f_{X,Y}(x,y)$ is the joint distribution function of *X* and *Y*.

## Appendix B Taylor approximation

### B.1 Selection probability approximation

Let *X* and *Y* be any random variables and *f* be an infinitely differentiable function of *X* and *Y*. We can approximate the expected value of *f*(*X*, *Y*) by performing a Taylor expansion:

$$\begin{aligned} {\mathbb {E}}\left[ f(X,Y)\right]= & {} {\mathbb {E}}\left[ f(\mu _X + (X-\mu _X), \mu _Y + (Y - \mu _Y))\right] \\\approx & {} {\mathbb {E}}\Bigg [f(\mu _X, \mu _Y)+f_X(\mu _X, \mu _Y)(X-\mu _X)+f_Y(\mu _X, \mu _Y)(Y-\mu _Y) \\&+f_{XY}(\mu _X, \mu _Y)(X-\mu _X)(Y-\mu _Y) \\&+\frac{1}{2}f_{XX}(\mu _X, \mu _Y)(X-\mu _X)^2+\frac{1}{2}f_{YY}(\mu _X, \mu _Y)(Y-\mu _Y)^2\Bigg ], \end{aligned}$$

where $\mu _X$ and $\mu _Y$ are the expected values of *X* and *Y*, respectively. Since ${\mathbb {E}}\left[ (X-\mu _X)\right] ={\mathbb {E}}\left[ (Y-\mu _Y)\right] =0$, and *X* and *Y* are independent, this simplifies to

$$\begin{aligned} {\mathbb {E}}\left[ f(\mu _X, \mu _Y)\right]&\approx f(\mu _X, \mu _Y) +\frac{1}{2}f_{XX}(\mu _X, \mu _Y)\sigma _X^2+\frac{1}{2}f_{YY}(\mu _X, \mu _Y)\sigma _Y^2, \end{aligned}$$

where $\sigma ^2_X$ and $\sigma ^2_Y$ are the variances of *X* and *Y*, respectively.

If we define $Y=\sum \limits _{j=1}^nf_j^R$ and $X=\sum \limits _{i=1}^mf_i^M$, then the selection probability is the expected value of a function of *X* and *Y*, $f(X,Y)=\frac{X}{X+Y}$, and we can approximate this as

$$\begin{aligned} {\mathbb {E}}\left[ \frac{X}{X+Y}\right] \approx \frac{\mu _X}{\mu _X+\mu _Y} + \frac{\mu _X}{(\mu _X+\mu _Y)^3}\sigma ^2_Y + \frac{\mu _X}{(\mu _X+\mu _Y)^3}\sigma ^2_X - \frac{1}{(\mu _X+\mu _Y)^2}\sigma ^2_X. \end{aligned}$$

Since $Y=\sum \limits _{j=1}^nf_j^R$ and $X=\sum \limits _{i=1}^mf_i^M$, we have $\mu _X=m\mu _M$, $\mu _Y=n\mu _R$, $\sigma ^2_X=m\sigma ^2_M$ and $\sigma ^2_Y=n\sigma ^2_R$, where $\mu _Z$ and $\sigma ^2_Z$ are mean and variance, respectively, for type $Z \in \{M,R\}$ (when the $f_j^R$ are independent for all *j* and the $f_i^M$ are independent for all *i*, which holds for within-generational variation).

### B.2 Critical variance

Assume we have *m* bet-hedgers in the selection group. We want to know when a bet-hedger would be have a higher relative strength in this scenario, and therefore we want to compare the selection probability $P(M|m \text { type } M \text { and } k-m \text { type } R)$ to the selection probability of a normal-type when there are *m* normal-types in the selection group, $P(R|k-m \text { type } M \text { and } m \text { type } R)=1-P(M|k-m \text { type } M \text { and } m \text { type } R)$. When these two probabilities are equal, the two types are equally favoured in such a scenario. Finding the variances for which these two are equal therefore gives the critical variances at which the bet-hedger becomes stronger in this scenario. Setting $P(M|m \text { type } M \text { and } n \text { type } R)=P(R|k-m \text { type } M \text { and } m \text { type } R)$ under the Taylor approximation we get

$$\begin{aligned}&\frac{m\mu _M}{(m\mu _M+n\mu _R)}+\frac{mn\mu _M}{(m\mu _M+n\mu _R)^3} \sigma ^2_R-\frac{m}{(m\mu _M+n\mu _R)^2}\sigma ^2_M +\frac{m^2\mu _M}{(m\mu _M+n\mu _R)^3}\sigma ^2_M\\&\quad = 1 - \frac{n\mu _M}{(n\mu _M+m\mu _R)}+\frac{nm\mu _M}{(n\mu _M+m\mu _R)^3}\sigma ^2_R\\&\qquad -\frac{n}{(n\mu _M+m\mu _R)^2}\sigma ^2_M+\frac{n^2\mu _M}{(n\mu _M+m\mu _R)^3}\sigma ^2_M \end{aligned}$$

which we can solve for $\sigma _R^2$ to obtain Eq. (5), which gives the critical normal-type variance at which the bet-hedging type is stronger, as a function of bet-hedger variance, when there are *m* bet-hedgers competing among *k* individuals.

## Appendix C Gamma distribution properties

### C.1 Calculating the upper bound

#### C.1.1 Case 1: $\sigma ^2_M = 0$

To calculate the upper bound exactly we wish the find the variation (which is equivalent to variance for the gamma distribution) at which the probability of selecting a bet-hedger to reproduce in the evenly-mixed scenario, i.e. *k*/2 bet-hedger versus *k*/2 normal-types, is equal to 1/2. We first consider the case where $\sigma ^2_M = 0$, so bet-hedger fitness is assumed constant rather than a random variable. In this case, selection probability is only a function of the normal-type fitness.

If we assume that the bet-hedger has constant fitness $\mu _M$ and that the normal-type fitness is drawn from a gamma distribution, $f_j^R \sim \Gamma (\mu _R/\theta ,\theta )$, then the sum of normal-type fitness is given by $Z \sim \Gamma (m\mu _R/\theta ,\theta )$, where $m=k/2$. To find the critical normal-type variation, we need to find the value of $\theta$ which satisfies

$$\begin{aligned} \int \limits _0^\infty \frac{m\mu _M}{m\mu _M+z}\frac{1}{\Gamma (\frac{m\mu _R}{\theta })\theta ^{\frac{m\mu _R}{\theta }}}z^{\frac{m\mu _R}{\theta }-1}e^{-\frac{z}{\theta }}dz=\frac{1}{2}. \end{aligned}$$

To solve this, we define

$$\begin{aligned} f(\theta )=\int \limits _0^\infty \frac{m\mu _M}{m\mu _M+z}\frac{1}{\Gamma (\frac{m\mu _R}{\theta })\theta ^{\frac{m\mu _R}{\theta }}}z^{\frac{m\mu _R}{\theta }-1}e^{-\frac{z}{\theta }}dz-\frac{1}{2}. \end{aligned}$$

Solving $f(\theta )=0$ analytically is challenging. However, for given values of $\theta$ it is easy to solve the integral numerically, since $m=k/2$, $\mu _M$ and $\mu _R$ are all known. Therefore we can construct a minimisation problem, where we aim the minimise the function $|f(\theta )|$. Since the integral is an increasing function of $\theta$, which we know from the definition of convex order, $f(\theta )$ is an increasing function and therefore $|f(\theta )|$ does not have local minima, and therefore minimising this function can be efficiently implemented in Matlab (or other language) to find the critical value for $\theta$.

#### C.1.2 Case 2: $\sigma ^2_M \ne 0$

Now we calculate the upper bound in the case where $\sigma ^2_M \ne 0$. Here, selection probability is a function of both bet-hedger and normal-type fitness. We assume that the sum of bet-hedger fitness is drawn from $X \sim \Gamma (m\mu _M/\theta _M,\theta _M)$ and normal-type fitness is drawn from $Y \sim \Gamma (m\mu _R/\theta _R,\theta _R)$. We are interested in pairs $(\theta _M,\theta _R)$ that satisfy

$$\begin{aligned} \int \limits _0^\infty \int \limits _0^\infty \frac{x}{x+y}\frac{1}{\Gamma (\frac{m\mu _M}{\theta _M})\theta _M^{\frac{m\mu _M}{\theta _M}}}x^{\frac{m\mu _M}{\theta _M}-1}e^{-\frac{x}{\theta _M}}\frac{1}{\Gamma (\frac{m\mu _R}{\theta _R})\theta _R^{\frac{m\mu _R}{\theta _R}}}y^{\frac{m\mu _R}{\theta _R}-1}e^{-\frac{y}{\theta _R}}dxdy=\frac{1}{2}. \end{aligned}$$

Defining

$$\begin{aligned} f(\theta _M,\theta _R) = \int \limits _0^\infty \int \limits _0^\infty \frac{x}{x+y}\frac{1}{\Gamma (\frac{m\mu _M}{\theta _M})\theta _M^{\frac{m\mu _M}{\theta _M}}}x^{\frac{m\mu _M}{\theta _M}-1}e^{-\frac{x}{\theta _M}}\frac{1}{\Gamma (\frac{m\mu _R}{\theta _R})\theta _R^{\frac{m\mu _R}{\theta _R}}}y^{\frac{m\mu _R}{\theta _R}-1}e^{-\frac{y}{\theta _R}}dxdy-\frac{1}{2}, \end{aligned}$$

such pairs are given by solutions to $f(\theta _M,\theta _R)=0$. Similarly to the previous case, this is challenging to solve analytically. However, this can again be solved efficiently using numerical minimisation. In particular, assuming either $\theta _M$ or $\theta _R$ is known, then this becomes a one dimensional function, which we will denote $g(\theta )$. As before, this will be an increasing function of the remaining $\theta$ term, so $|g(\theta )|$ has no local minima, and the function can easily be minimised in Matlab to find the critical $\theta$. If $\theta _M$ is known this will give the corresponding critical $\theta _R$, or if $\theta _R$ is known then this will give $\theta _M$.

### C.2 Linear upper bound

#### C.2.1 Case 1: $\sigma ^2_M = 0$

Our results from the Taylor approximation suggest that the upper bound on the overall critical normal-type variance required for the bet-hedger to be favoured is given by the evenly mixed scenario, i.e. $m=n=k/2$. Here we explore how changing selection group size impacts the critical normal-type variance for this scenario (and therefore the upper bound) using the exact selection probability, when normal-type fitness is drawn from a gamma distribution. For the gamma distribution convex order reduces to ordering the variance of the distributions, so we can represent the critical normal-type variation using the variance. We first assume that bet-hedgers have constant fitness equal to $\mu _M$ and normal-types have fitness $f^R_j \sim \Gamma (\mu _R/\theta ,\theta )$, such that the normal-type have mean fitness $\mu _R$ and variance in fitness given by $\mu _R\theta$. We consider this case first since the results from the Taylor approximation suggest that in this case critical normal-type variation will increase linearly with selection group size. The selection probability of a bet-hedger is given by

$$\begin{aligned} P(M|m)={\mathbb {E}}\left[ \frac{m\mu _M}{m\mu _M+\sum \limits _{j=1}^mf^R_j}\right] . \end{aligned}$$

Since each $f^R_j$ is independent and identically distributed we can write $Z=\sum \nolimits _{j=1}^mf^R_j \sim \Gamma (m\mu _R/\theta ,\theta )$. Therefore, we can now write the selection probability as

$$\begin{aligned} P(M|m)={\mathbb {E}}\left[ \frac{m\mu _M}{m\mu _M+Z}\right] =\int \limits _0^\infty \frac{m\mu _M}{m\mu _M+z}\frac{1}{\Gamma (\frac{m\mu _R}{\theta })\theta ^{\frac{m\mu _R}{\theta }}}z^{\frac{m\mu _R}{\theta }-1}e^{-\frac{z}{\theta }}dz. \end{aligned}$$

The critical normal-type variance for the evenly mixed scenario, $\sigma _R^2({k,m})=\mu _R\theta _{k,m}$, needs to satisfy

$$\begin{aligned} \int \limits _0^\infty \frac{m\mu _M}{m\mu _M+z}\frac{1}{\Gamma (\frac{m\mu _R}{\theta _{k,m}})\theta _{k,m}^{\frac{m\mu _R}{\theta _{k,m}}}}z^{\frac{m\mu _R}{\theta _{k,m}}-1}e^{-\frac{z}{\theta _{k,m}}}dz=\frac{1}{2}, \end{aligned}$$

where $m=n=k/2$. Changing the selection group size to *hk*, we are interested in the selection probability with $x=hm$ bet-hedgers. The critical normal-type variance $\mu _R\theta _{hk,hm}$ needs to satisfy

$$\begin{aligned} \int \limits _0^\infty \frac{hm\mu _M}{hm\mu _M+z}\frac{1}{\Gamma (\frac{hm\mu _R}{\theta _{hk,hm}})\theta _{hk,hm}^{\frac{hm\mu _R}{\theta _{hk,hm}}}}z^{\frac{hm\mu _R}{\theta _{hk,hm}}-1}e^{-\frac{z}{\theta _{hk,hm}}}dz=\frac{1}{2}. \end{aligned}$$

Using a change of variable $z'=z/h$ this becomes

$$\begin{aligned} \int \limits _0^\infty \frac{hm\mu _M}{hm\mu _M+hz'}\frac{1}{\Gamma (\frac{hm\mu _R}{\theta _{hk,hm}})\theta _{hk,hm}^{\frac{hm\mu _R}{\theta _{hk,hm}}}}(hz')^{\frac{hm\mu _R}{\theta _{hk,hm}}-1}e^{-\frac{hz'}{\theta _{hk,hm}}}hdz'=\frac{1}{2}. \end{aligned}$$

Now if we set $\sigma _{k,hm}^2=d\theta _{k,hm}=dh\theta _{k,m}=h\sigma _{k,m}^2$ then this reduces to

$$\begin{aligned} \int \limits _0^\infty \frac{m\mu _M}{m\mu _M+z'}\frac{1}{\Gamma (\frac{m\mu _R}{\theta _{k,m}})\theta _{k,m}^{\frac{m\mu _R}{\theta _{k,m}}}}(z')^{\frac{m\mu _R}{\theta _{k,m}}-1}e^{-\frac{z'}{\theta _{k,m}}}dz'=\frac{1}{2}, \end{aligned}$$

which we know holds, and therefore for the gamma distribution the critical normal-type variance in the evenly mixed scenario is proportional to the size of the selection group.

#### C.2.2 Case 2: $\sigma ^2_M \ne 0$

In the case where $\sigma _M^2 \ne 0$, the critical normal-type variation is no longer proportional to the selection group size. However, in the Taylor approximation we showed that the critical normal-type variation is a linear function of both $\sigma _M^2$ and *k*, so that if $\sigma _M^2$ and *k* increase by a given proportion, so does the critical variation. Here we show that this also holds when the fitnesses are drawn from gamma distributions.

If the bet-hedger and normal-type strategies are balanced, then we have a pair $(\theta _M,\theta _R)$ that satisfies

$$\begin{aligned} \int \limits _0^\infty \int \limits _0^\infty \frac{x}{x+y}\frac{1}{\Gamma \left( \frac{m\mu _M}{\theta _M}\right) \theta _M^{\frac{m\mu _M}{\theta _M}}}x^{\frac{m\mu _M}{\theta _M}-1} e^{-\frac{x}{\theta _M}}\frac{1}{\Gamma \left( \frac{m\mu _R}{\theta _R}\right) \theta _R^{\frac{m\mu _R}{\theta _R}}}y^{\frac{m\mu _R}{\theta _R}-1} e^{-\frac{y}{\theta _R}}dxdy=\frac{1}{2}. \end{aligned}$$

where $m=k/2$. Changing the selection group size to *hk*, we are interested in the selection probability with $m'=hm$ bet-hedgers. To keep the balance, we need a pair $(\theta _M',\theta _R')$ that satisfies

$$\begin{aligned}&\int \limits _0^\infty \int \limits _0^\infty \frac{x}{x+y}\frac{1}{\Gamma \left( \frac{hm\mu _M}{\theta _M'}\right) \theta _M'^{\frac{hm\mu _M}{\theta _M'}}} x^{\frac{hm\mu _M}{\theta _M'}-1} e^{-\frac{x}{\theta _M'}}\\&\quad \times \frac{1}{\Gamma \left( \frac{hm\mu _R}{\theta _R'}\right) \theta _R'^{\frac{hm\mu _R}{\theta _R'}}} y^{\frac{hm\mu _R}{\theta _R'}-1}e^{-\frac{y}{\theta _R'}}dxdy=\frac{1}{2}. \end{aligned}$$

Based on the Taylor approximation, trying $(\theta _M',\theta _R') = h \times (\theta _M,\theta _R)$ gives

$$\begin{aligned}&\int \limits _0^\infty \int \limits _0^\infty \frac{x}{x+y} \frac{1}{\Gamma \left( \frac{hm\mu _M}{h\theta _M}\right) (h\theta _M)^{\frac{hm\mu _M}{h\theta _M}}}x^{\frac{hm\mu _M}{h\theta _M}-1} e^{-\frac{x}{h\theta _M}}\\&\quad \times \frac{1}{\Gamma \left( \frac{hm\mu _R}{h\theta _R}\right) (h\theta _R)^{\frac{hm\mu _R}{h\theta _R}}} y^{\frac{hm\mu _R}{h\theta _R}-1}e^{-\frac{y}{h\theta _R}}dxdy=\frac{1}{2}. \end{aligned}$$

Using a change of variable $x = hx'$ and $y = hy'$ this becomes

$$\begin{aligned}&\int \limits _0^\infty \int \limits _0^\infty \frac{hx'}{hx'+hy'} \frac{1}{\Gamma \left( \frac{hm\mu _M}{h\theta _M}\right) (h\theta _M)^{\frac{hm\mu _M}{h\theta _M}}}(hx')^{\frac{hm\mu _M}{h\theta _M}-1} e^{-\frac{hx'}{h\theta _M}}\\&\quad \times \frac{1}{\Gamma \left( \frac{hm\mu _R}{h\theta _R}\right) (h\theta _R)^{\frac{hm\mu _R}{h\theta _R}}}(hy')^{\frac{hm\mu _R}{h\theta _R}-1}e^{-\frac{hy'}{h\theta _R}}h^2dx'dy'=\frac{1}{2}, \end{aligned}$$

which reduces to

$$\begin{aligned}&\int \limits _0^\infty \int \limits _0^\infty \frac{x'}{x'+y'}\frac{1}{\Gamma \left( \frac{m\mu _M}{\theta _M}\right) (\theta _M)^{\frac{m\mu _M}{\theta _M}}}(x')^{\frac{m\mu _M}{\theta _M}-1}e^{-\frac{x'}{\theta _M}}\\&\quad \times \frac{1}{\Gamma \left( \frac{m\mu _R}{\theta _R}\right) (\theta _R)^{\frac{m\mu _R}{\theta _R}}}(y')^{\frac{m\mu _R}{\theta _R}-1}e^{-\frac{y'}{\theta _R}}dx'dy'=\frac{1}{2}. \end{aligned}$$

We know this equation holds, and therefore for the gamma distribution in the evenly-mixed case, if the selection group size undergoes a proportional increase, the dynamics will be balanced if both variances increase by this proportion.

## Declarations

### Data and materials

This paper makes extensive use of stochastic simulations. Code to generate the results shown in this paper, as well as general code to simulate the model, is available as supplementary material, and can also be found at https://github.com/OvertonC/bet-hedging.

## References

[CR1] Altrock PM, Traulsen A (2009). Deterministic evolutionary game dynamics in finite populations. Phys Rev E.

[CR2] Antal T, Redner S, Sood V (2006). Evolutionary dynamics on degree-heterogeneous graphs. Phys Rev Lett.

[CR3] Argasinski K, Broom M (2013). Ecological theatre and the evolutionary game: how environmental and demographic factors determine payoffs in evolutionary games. J Math Biol.

[CR4] Beaumont HJE, Gallie J, Kost C, Ferguson GC, Rainey PB (2009). Experimental evolution of bet hedging. Nature.

[CR5] Broom M, Rychtář J (2008). An analysis of the fixation probability of a mutant on special classes of non-directed graphs. Proc R Soc A Math Phys Eng Sci.

[CR6] Broom M, Hadjichrysanthou C, Rychtář J (2010). Evolutionary games on graphs and the speed of the evolutionary process. Proc R Soc A Math Phys Eng Sci.

[CR7] Cohen D (1966). Optimizing reproduction in a randomly varying environment. J Theor Biol.

[CR8] Courtney SP (1986) Why insects move between host patches: some comments on ‘risk-spreading’. *Oikos*, pp 112–114

[CR9] Czuppon P, Traulsen A (2018). Fixation probabilities in populations under demographic fluctuations. J Math Biol.

[CR10] Erdős P, Rényi A (1960). On the evolution of random graphs. Publ Math Inst Hung Acad Sci.

[CR11] Fox CW, Rauter CM (2003). Bet-hedging and the evolution of multiple mating. Evol Ecol Res.

[CR12] Frank SA, Slatkin M (1990). Evolution in a variable environment. Am Nat.

[CR13] Giaimo S, Arranz J, Traulsen A (2018). Invasion and effective size of graph-structured populations. PLoS Comput Biol.

[CR14] Gillespie JH (1973). Natural selection with varying selection coefficients-a haploid model. Genet Res.

[CR15] Gillespie JH (1974). Natural selection for within generation variance in offspring number. Genetics.

[CR16] Gravenmier CA, Siddique M, Gatenby RA (2018). Adaptation to stochastic temporal variations in intratumoral blood flow: the warburg effect as a bet hedging strategy. Bull Math Biol.

[CR17] Hindersin L, Traulsen A (2015). Most undirected random graphs are amplifiers of selection for birth-death dynamics, but suppressors of selection for death-birth dynamics. PLoS Comput Biol.

[CR18] Hindersin L, Werner B, Dingli D, Traulsen A (2016). Should tissue structure suppress or amplify selection to minimize cancer risk?. Biol Direct.

[CR19] Hopper KR (1999). Risk-spreading and bet-hedging in insect population biology. Ann Rev Entomol.

[CR20] Hopper KR, Rosenheim JA, Prout T, Oppenheim SJ (2003). Within-generation bet hedging: a seductive explanation?. Oikos.

[CR21] Lehmann L, Balloux F (2007). Natural selection on fecundity variance in subdivided populations: kin selection meets bet-hedging. Genetics.

[CR22] Levy SF, Ziv N, Siegal ML (2012). Bet hedging in yeast by heterogeneous, age-correlated expression of a stress protectant. PLoS Biol.

[CR23] Lieberman E, Hauert C, Nowak MA (2005). Evolutionary dynamics on graphs. Nature.

[CR24] Masuda N (2009). Directionality of contact networks suppresses selection pressure in evolutionary dynamics. J Theor Biol.

[CR25] Moran PAP (1958). Random processes in genetics. Math Proc Cambridge Philos Soc.

[CR26] Ohtsuki H, Hauert C, Lieberman E, Nowak MA (2006). A simple rule for the evolution of cooperation on graphs and social networks. Nature.

[CR27] Olofsson H, Ripa J, Jonzén N (2009). Bet-hedging as an evolutionary game: the trade-off between egg size and number. Proc R Soc B Biol Sci.

[CR28] Philippi T (1993). Bet-hedging germination of desert annuals: variation among populations and maternal effects in lepidium lasiocarpum. Am Nat.

[CR29] Renton J, Page KM (2019). Evolution of cooperation in an epithelium. J R Soc Interface.

[CR30] Rice SH (2008). A stochastic version of the price equation reveals the interplay of deterministic and stochastic processes in evolution. BMC Evol Biol.

[CR31] Rice SH, Papadopoulos A (2009). Evolution with stochastic fitness and stochastic migration. PLoS ONE.

[CR32] Roff DA (2008). Defining fitness in evolutionary models. J Genet.

[CR33] Root RB, Kareiva PM (1984). The search for resources by cabbage butterflies (pieris rapae): ecological consequences and adaptive significance of markovian movements in a patchy environment. Ecology.

[CR34] Sarhan A, Kokko H (2007). Multiple mating in the glanville fritillary butterfly: A case of within-generation bet hedging?. Evolution.

[CR35] Seger J (1987). What is bet-hedging?. Oxford Surv Evol Biol.

[CR36] Shaked M, Shanthikumar JG (2007). Stochastic orders.

[CR37] Shpak M (2005). Evolution of variance in offspring number: the effects of population size and migration. Theory Biosci.

[CR38] Shpak M, Proulx SR (2007). The role of life cycle and migration in selection for variance in offspring number. Bull Math Biol.

[CR39] Starrfelt J, Kokko H (2012). Bet-hedging-a triple trade-off between means, variances and correlations. Biol Rev.

[CR40] Stumpf MPH, Laidlaw Z, Jansen VAA (2002). Herpes viruses hedge their bets. Proc Nat Acad Sci.

[CR41] Traulsen A, Hauert C (2010). Stochastic evolutionary game dynamics. Rev Nonlinear Dyn Complex.

[CR42] Tufto J (2015). Genetic evolution, plasticity, and bet-hedging as adaptive responses to temporally autocorrelated fluctuating selection: a quantitative genetic model. Evolution.

[CR43] Venable DL (2007). Bet hedging in a guild of desert annuals. Ecology.

[CR44] Ward SA, Dixon AFG (1984) Spreading the risk, and the evolution of mixed strategies: seasonal variation in aphid reproductive biology. In: Advances in Invertebrate Reproduction 3: Proceedings of 3rd international symposium, international society of invertebrate reproduction. Elsevier Science Publishers, Amsterdam

[CR45] Watson PJ (1991). Multiple paternity as genetic bet-hedging in female sierra dome spiders, linyphia litigiosa (linyphiidae). Anim Behav.

[CR46] Wild G, Taylor PD (2004). Fitness and evolutionary stability in game theoretic models of finite populations. Proc R Soc Lond Ser B Biol Sci.

[CR47] Wilkinson RR, Sharkey KJ (2018). Impact of the infectious period on epidemics. Phys Rev E.

[CR48] Yasui Y (2001). Female multiple mating as a genetic bet-hedging strategy when mate choice criteria are unreliable. Ecol Res.

[CR49] Yasui Y, Garcia-Gonzalez F (2016). Bet-hedging as a mechanism for the evolution of polyandry, revisited. Evolution.

[CR50] Yasui Y, Yoshimura J (2018). Bet-hedging against male-caused reproductive failures may explain ubiquitous cuckoldry in female birds. J Theor Biol.

